# Tributyltin Inhibits Neural Induction of Human Induced Pluripotent Stem Cells

**DOI:** 10.1038/s41598-018-30615-2

**Published:** 2018-08-14

**Authors:** Shigeru Yamada, Yusuke Kubo, Daiju Yamazaki, Yuko Sekino, Yoko Nomura, Sachiko Yoshida, Yasunari Kanda

**Affiliations:** 1 Division of Pharmacology, National Institute of Health Sciences, Kanagawa, Japan; 2 Graduate School of Pharmaceutical Sciences, The University of Tokyo, Tokyo, Japan; 3 Department of Psychology, Queens College and The Graduate Center, New York, USA; 4 Department of Environmental and Life Sciences, Toyohashi University of Technology, Toyohashi, Japan; 5 Pharmacological Evaluation Institute of Japan (PEIJ), Kanagawa, Japan

## Abstract

Tributyltin (TBT), one of the organotin compounds, is a well-known environmental pollutant. In our recent study, we reported that TBT induces mitochondrial dysfunction, in human-induced pluripotent stem cells (iPSCs) through the degradation of mitofusin1 (Mfn1), which is a mitochondrial fusion factor. However, the effect of TBT toxicity on the developmental process of iPSCs was not clear. The present study examined the effect of TBT on the differentiation of iPSCs into the ectodermal, mesodermal, and endodermal germ layers. We found that exposure to nanomolar concentration of TBT (50 nM) selectively inhibited the induction of iPSCs into the ectoderm, which is the first step in neurogenesis. We further assessed the effect of TBT on neural differentiation and found that it reduced the expression of several neural differentiation marker genes, which were also downregulated by Mfn1 knockdown in iPSCs. Taken together, these results indicate that TBT induces developmental neurotoxicity via Mfn1-mediated mitochondrial dysfunction in iPSCs.

## Introduction

Organotin compounds, such as tributyltin (TBT), are typical environmental contaminants and endocrine disruptive chemicals, causing various developmental defects including increased fetal mortality, decreased fetal birth weight, behavioral abnormalities, and teratogenicity in the offspring of rats^1,2^. Although the use of TBT is currently restricted, the presence of butyltin compounds, including TBT, has been reported at nanomolar levels (50–400 nM) in human blood^3^. In the present study, we set out to elucidate the mechanisms through which nanomolar TBT levels cause developmental toxicity. We used undifferentiated normal stem cells as the most suitable platform for differentiation studies.

Several studies have revealed the cytotoxic effects of nanomolar concentrations of TBT in stem (like) cells. For example, TBT is known to activate retinoid X receptor (RXR) and/or peroxisome proliferator-activated receptor γ (PPARγ). These genomic transcriptional activations result in developmental effects, such as the imposex in many marine species^4^ and the enhancement of adipocyte differentiation in adipose-derived stromal stem cells (ADSCs)^5^. Moreover, transcriptome analysis after induction of TBT-dependent apoptosis revealed changes in the expression levels of genes involved in Ca^2+^ mobilization, retinoic acid signaling, and apoptosis^6^. In addition to the genomic effects, we previously found the non-genomic action of TBT. TBT reduced intracellular ATP levels by targeting glycolysis and mitochondrial systems, and inhibited the growth of human embryonic carcinoma NT2/D1 cells^7–10^. We also found that TBT induced the growth inhibition of human induced pluripotent stem cells (iPSCs) through mitochondrial dysfunction, such as decreased ATP levels, depolarization of mitochondrial membrane potential (MMP) and mitochondrial fragmentation, via the degradation of mitochondrial fusion factor, mitofusin1 (Mfn1)^11^.

Mitochondria are dynamic organelles that continuously undergo fusion and fission events. Mitochondrial fusion is regulated by the fusion factors Mfn1, Mfn2, and optic atrophy 1 (Opa1)^12,13^, and produces elongated or tubular mitochondria, which facilitate the exchange and equal distribution of metabolites between mitochondria^14^. Mitochondrial fission is regulated by fission factors such as fission protein 1 (Fis1) and dynamin-related protein 1 (Drp1)^15,16^, and not only facilitates the generation of healthy new mitochondria but also allows the segregation of non-functional mitochondria^17^. Thus, these morphological changes maintain mitochondrial quality, which is responsible for cellular energy supply, by allowing impaired mitochondria to be recycled^14,17^. Therefore, mitochondrial dynamics are necessary for cell survival as well as adaptation to changing conditions needed for cell growth^18^. Several studies have shown the relationship between mitochondrial fragmentation and cellular and neurodevelopmental defects. For example, Mfn1 knockout mice show developmental delay at the midgestational embryonic stage and ultimately die^19^. In addition, embryonic fibroblasts from these knockout mice display distinct types of fragmented mitochondria, which is a phenotypical characteristic of a severe reduction in mitochondrial fusion^19^. Based on these findings, we hypothesized that nanomolar TBT could also affect the developmental process of iPSCs, which can differentiate into somatic cells from three developmental germ layers (ectoderm, mesoderm, endoderm)^20^.

In the present study, we investigated the effect of TBT on the differentiation of iPSCs into germ layers as a model of human organ development^21^. Our results showed that 50 nM TBT selectively suppressed the induction of iPSCs into the ectoderm during neurogenesis. Moreover, TBT reduced the expression of several neural differentiation marker genes, which were also downregulated by Mfn1 knockdown. These data suggest that TBT-induced neurodevelopmental toxicity involves Mfn1-mediated mitochondrial dysfunction in human iPSCs, without affecting mesodermal and endodermal inductions.

## Results

### Effect of TBT on Differentiation of iPSCs into Three Germ Layers

**Figure 1:**
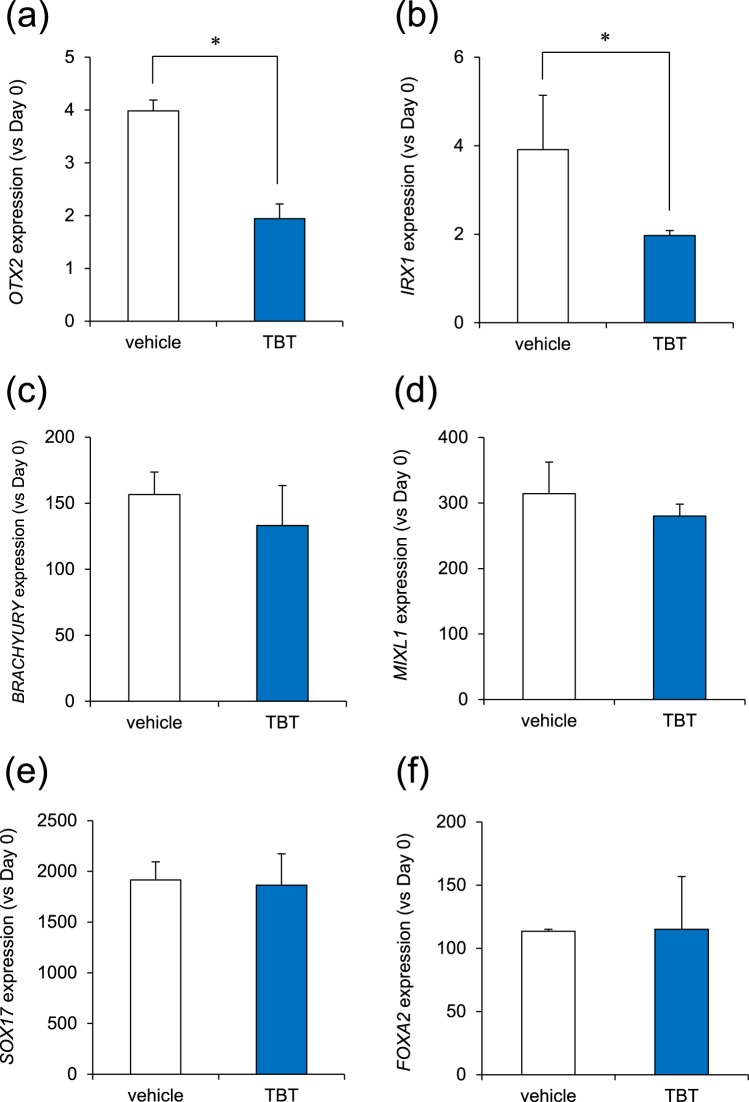
TBT inhibits ectodermal induction of iPSCs. (**a**,**b**) Ectodermal induction was initiated after exposure to 50 nM TBT for 24 h. Cells were continuously exposed to TBT throughout the induction. At day 4 after the induction, the expression of ectodermal markers (*OTX2*, *IRX1*) was examined using real-time PCR analysis. (**c**,**d**) Mesodermal induction was initiated after exposure to 50 nM TBT for 24 h. Cells were continuously exposed to TBT throughout the induction. At day 1 after the induction, the expression of mesoderm markers (*BRACHYURY*, *MIXL1*) was examined using real-time PCR analysis. (**e**,**f**) Endodermal induction was initiated after exposure to 50 nM TBT for 24 h. Cells were continuously exposed to TBT throughout the induction. At day 4 after the induction, the expression of endoderm markers (*SOX17*, *FOXA2*) was examined using real-time PCR analysis. Each bar represents the mean ± SD from three independent experiments. *P < 0.05.

To examine the effects of TBT on fetal development, we studied its effects on the differentiation of iPSCs into ectodermal, mesodermal, and endodermal germ layers. In ectoderm induction, we found that treatment with 50 nM TBT significantly reduced the gene expression of the *OTX2* marker that regulates neurogenesis^22^ (Fig. 1a). We also found that TBT reduced another ectodermal marker *IRX1* expression^23^ (Fig. 1b). In contrast, TBT had little effect on the inductions of mesodermal (*BRACHYURY*, *MIXL1*) and endodermal (*SOX17*, *FOXA2*) markers^24–27^ (Fig. 1c–f). TBT at 50 nM has the ability to bind to PPARγ with a higher affinity than that of the intrinsic ligands, and these genomic transcriptional activations have been reported to mediate neurodevelopmental defects^5^. To investigate the molecular mechanisms by which TBT inhibits ectodermal induction, we examined the effect of the PPARγ agonist rosiglitazone (RGZ), which had been confirmed in our previous report, as having agonistic effects on PPARγ^10^. We found that RGZ did not reduce *OTX2* expression (Fig. S1). Taken together, these data suggest that TBT inhibits ectodermal induction in iPSCs regardless of PPARγ activation.

### Effect of TBT on Neural Differentiation of iPSCs

**Figure 2:**
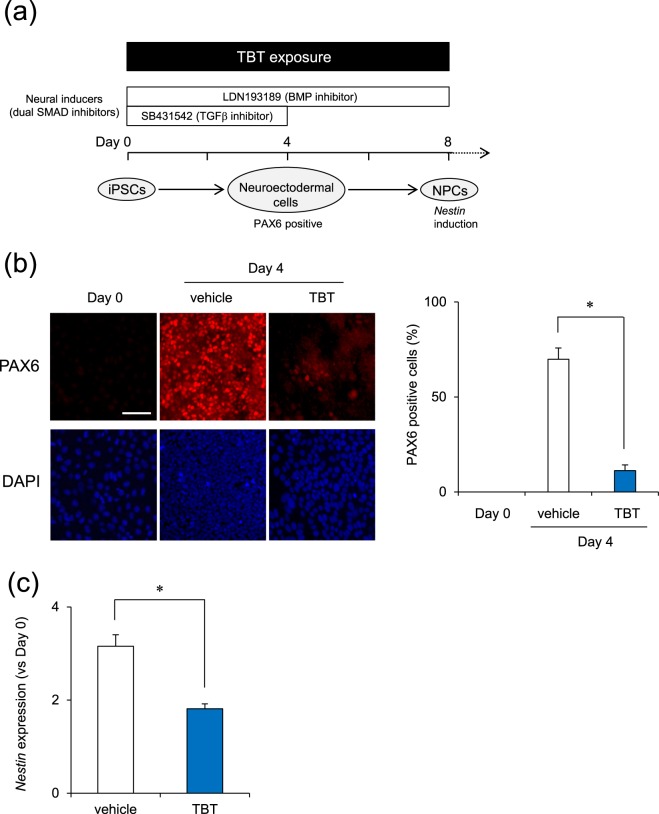
TBT inhibits neural differentiation of iPSCs. (**a**) Schematic time course of NPC induction from iPSCs using dual SMAD inhibition. Neural induction was initiated after exposure to 50 nM TBT for 24 h. Cells were continuously exposed to TBT throughout neural differentiation. (**b**) At day 4 after neural induction, the expression of neuroectodermal marker, PAX6, was observed by immunocytochemistry using anti-PAX6 antibodies. Nuclei were counterstained with DAPI. PAX6 positive nuclei were counted using the ImageJ software. Bar = 100 μm. (**c**) At day 8 after neural induction, the expression of NPC marker, *Nestin*, was examined using real-time PCR analysis. Each bar represents the mean ± SD from three independent experiments. *P < 0.05.

The production of ectodermal germ layer is the first step in neurogenesis^28^. Next, to examine the effects of TBT on neural differentiation, we performed the differentiation into neural progenitor cells (NPCs) from iPSCs (Fig. 2a). There has been reported for neural differentiation methods from iPSCs based on different protocols, such as neural induction through embryoid body formation^29^, inhibition of TGF-β and BMP signaling pathways (dual SMAD inhibition)^28^, or forced expression of neurogenin-2 with puromycin selection^30^. We chose dual SMAD inhibition protocol, because it is simple, non-viral, low-priced, non-time consuming, highly efficient, reproducible and similar to *in vivo* neurogenic processes among these methods. We examined the expression of several neural differentiation markers. Immunocytochemical analysis showed strong expression levels of PAX6, a marker of neuroectoderm^28^, by day 4 compared to that on day 0 while TBT exposure significantly decreased the percentage of PAX6 positive cells at day 4 (Fig. 2b). In addition, real-time PCR analysis revealed that TBT significantly downregulated the expression of *Nestin* (day 8), which is a marker of NPCs^31^ (Fig. 2c). These data suggest that TBT has an inhibitory effect on the neural induction of iPSCs.

### Effect of Mfn1 Knockdown on Neural Differentiation of iPSCs

**Figure 3:**
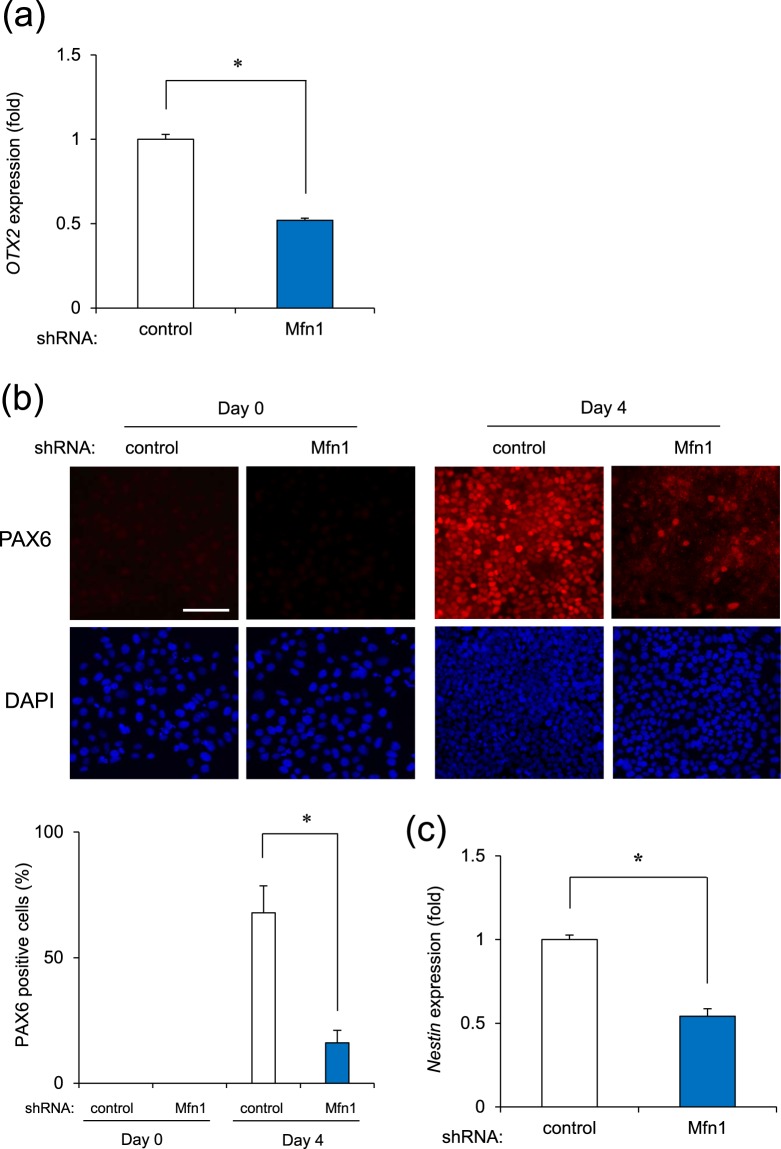
Mfn1 knockdown inhibits neural differentiation of iPSCs. (**a**–**c**) Cells were infected with lentiviruses containing a vector encoding shRNA directed against Mfn1 or scrambled sequence shRNA (control) for 24 h. Infected cells were selected using puromycin (1 μg/ml) for 24 h and cultured for an additional 72 h before neural differentiation. (**a**) The expression of *OTX2* (day 2) was examined with real-time PCR. (**b**) The expression of PAX6 (day 4) was observed by immunocytochemistry using anti-PAX6 antibodies. Nuclei were counterstained with DAPI. PAX6 positive nuclei were counted using the ImageJ software. Bar = 100 μm. (**c**) The expression of *Nestin* (day 8) was examined with real-time PCR. Each bar represents the mean ± SD from three independent experiments. *P < 0.05.

We previously found that TBT induced mitochondrial dysfunction by degrading Mfn1^11^. In addition, as described above, Mfn1 is reported to be involved in neural development^19^. To investigate the involvement of Mfn1 in the effects of TBT on neural induction, we performed a knockdown (KD) of Mfn1 using lentivirus-delivered shRNAs. Our previous study showed that the KD was selective for Mfn1 and not Mfn2, with an efficiency of approximately 70%^32^. The Mfn1 KD cells were used to perform neural induction. We found that Mfn1 KD significantly reduced the gene expression of *OTX2* at day 2 after neural induction (Fig. 3a). Immunocytochemical analysis revealed a strong expression of PAX6 by day 4 after neural induction in the control cells while Mfn1 KD significantly reduced the percentage of PAX6 positive cells at day 4 (Fig. 3b). We further found that Mfn1 KD significantly decreased the gene expression of *Nestin* at day 8 (Fig. 3c). These data suggest that Mfn1 is involved in the TBT-mediated negative effects on neural induction of iPSCs.

### Negative Regulation of Neural Induction by TBT Exposure

**Figure 4:**
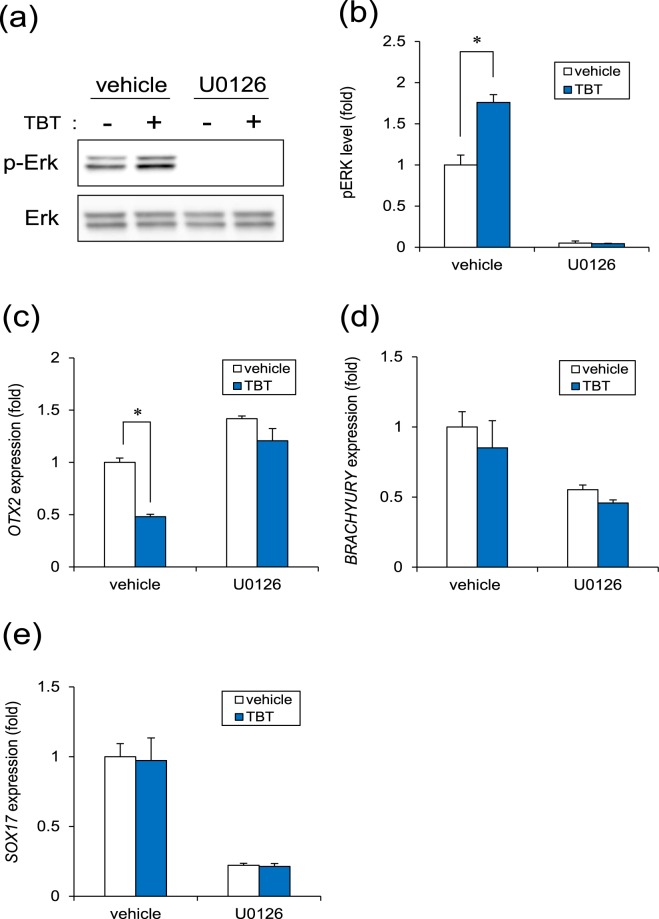
TBT negatively regulates neural induction. (**a**) Cells were exposed to TBT (50 nM) or TBT + U0126 (5 μM) for 24 h. ERK phosphorylation was analyzed by western blotting using anti-phospho-ERK antibodies. Cropped blots were shown and the full-length blots were indicated in Supplementary Fig. 3. (**b**) Relative densities of bands were quantified using ImageJ software. Relative changes in expression were determined by normalization to total ERK protein level. (**c**) At day 2 after ectodermal (neural) induction with TBT or TBT + U0126, the expression of *OTX2* gene was analyzed using real-time PCR. (**d**) At day 1 after mesodermal induction with TBT or TBT + U0126, the expression of *BRACHYURY* gene was analyzed using real-time PCR. (**e**) At day 4 after endoderm induction with TBT or TBT + U0126, the expression of *SOX17* gene was analyzed using real-time PCR. Each bar represents the mean ± SD from three independent experiments. *P < 0.05.

A previous report indicates that Mfn1 directly binds Ras and Raf, thereby inhibiting Ras-Raf-ERK signaling, as determined using biochemical analysis^33,34^. Thus, ERK has been reported to be activated after depletion of Mfn1^35^. Moreover, ERK signaling is known to inhibit neural induction via *OTX2* silencing in human embryonic stem cells^36^. Therefore, we focused on investigating the involvement of ERK signaling in the effect of TBT on neural induction. We found that TBT exposure significantly increased basal ERK phosphorylation levels, while this effect was neglected by treatment with the ERK inhibitor U0126 (Fig. 4a and b). To further investigate whether *OTX2* downregulation in TBT-exposed cells was mediated by ERK signaling, we examined the effect of U0126 on *OTX2* expression. Only U0126 treatment increased *OTX2* expression during ectodermal induction (Fig. 4c), suggesting that ERK signaling negatively regulated neural induction. In contrast, U0126 suppressed the expressions of *BRACHYURY* and *SOX17* marker genes in mesodermal and endodermal induction respectively (Fig. 4d and e), supporting previous reports that ERK signaling contributes to mesodermal and endodermal differentiation^37,38^. U0126 quenched the negative effect of TBT on *OTX2* gene expression (Fig. 4c). In the differentiation into three germ layers, we confirmed the Mfn1 downregulation in TBT-treated groups (Fig. S2). These data suggest that TBT prevents neural (ectodermal) induction via ERK phosphorylation and subsequent *OTX2* downregulation.

## Discussion

**Figure 5:**
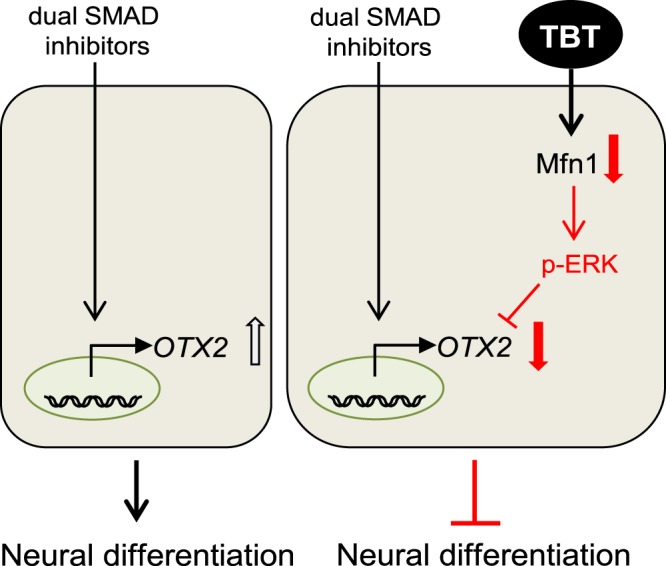
Proposed mechanism of TBT-induced developmental neurotoxicity in human iPSCs. TBT exposure causes Mfn1 reduction, which induces mitochondrial dysfunction, including mitochondrial fragmentation and decreased ATP levels. Mitochondrial dysfunction subsequently evokes ERK phosphorylation, leading to the suppression of *OTX2*, which is an early marker of neurogenesis.

In the present study, we demonstrated that exposure to 50 nM TBT inhibited ectoderm induction by suppression of *OTX2* in iPSCs. In addition, the negative effect of TBT on neurogenesis was likely mediated by Mfn1 degradation, followed by ERK phosphorylation. Based on the data obtained in our study, we propose a mechanism by which the TBT-induced developmental neurotoxicity is mediated by mitochondrial dysfunction (Fig. 5).

We showed that TBT selectively inhibited ectodermal induction during the differentiation of iPSCs into the three germ layers (Fig. 1). A previous report suggests the presence of organotin compounds such as TBT, at concentrations between 50 and 400 nM in human blood^3^. Thus, the concentration (50 nM) of TBT used in our studies are relevant to human exposure levels. Moreover, the inhibitory effect of TBT on ectodermal induction was also supported by the knockdown of Mfn1, which mimics the effect of TBT on Mfn1^11^. In contrast, TBT did not affect mesodermal or endodermal induction of iPSCs. A previous report has shown that the ablation of Mfn1 in the embryonic mouse heart, which is derived from embryonic mesodermal germ layer, arrests its development^39^. Therefore, TBT may affect the differentiation processes after the mesodermal germ layer, which in turn may potentially lead to cardiomyocyte. Further studies are needed to elucidate whether TBT actions contain the stage selectivity in the differentiation into mesodermal- or endodermal-derived cells.

We demonstrated that TBT negatively affected the levels of *OTX2* in the neural differentiation (Fig. 1), which acts as a transcriptional regulator during forebrain development in vertebrates^40^. In addition, the targeted disruption of PAX6 in rodents led to the loss of anterior neural tissues^41^, suggesting a central role for PAX6 in forebrain development. TBT exposure causes decreases in forebrain weight with a reduction of synaptogenic markers in the developing rat brain^42^. These TBT-induced defects of the forebrain may be caused by transcriptional silencing of anterior neural markers, such as OTX2 and PAX6, during early neurogenesis. As a neural progenitor marker, *Nestin* was downregulated during the neural differentiation of iPSCs exposed to TBT, and further studies in NPCs are required to determine whether TBT affects their differentiation into specific neuronal subtypes, e.g., glutamatergic, GABAergic, or cholinergic.

We further demonstrated that the negative effect of TBT on neurogenesis was probably mediated by Mfn1 degradation, followed by ERK phosphorylation (Fig. 4). A previous report indicates that Mfn1 directly binds Ras and Raf, thereby inhibiting Ras-Raf-ERK signaling, as determined using biochemical analysis^33,34^. Thus, Mfn1 reduction by TBT or shRNA could reverse the inhibition of ERK signaling. The mobilization of Ca^2+^ from intracellular stores including the mitochondria was reported to result in the phosphorylation of MAPKs, the authors stating that the process was suppressed by the chelation of intracellular Ca^2+^ in human T lymphoblastoid cells^43^. The established mitochondrial uptake of any Ca^2+^ that accumulated in the cytosol was shown to be dependent on MMP^44^ with the mitochondrial dysfunction induced by TBT exposure probably causing an overload of Ca^2+^, which resulted in ERK activation. Moreover, the ERK signaling was reported to inhibit neural induction by silencing *PAX6* through upregulation of the stemness factors *NANOG/OCT4* and downregulation of *OTX2*^36^. Thus, TBT-induced ERK phosphorylation could downregulate *PAX6* expression by the suppression of *OTX2* in mediating its developmental neurotoxicity in iPSCs. Moreover, ERK signaling evoked by TBT may affect the expression of other *PAX6* regulatory factors such as *NANOG/OCT4*. In future studies, it would be expedient for us to further investigate the mechanisms underlying the TBT-induced negative regulation of neural induction via ERK.

In summary, our results demonstrate a novel mechanism underlying the cytotoxicity and neurodevelopmental toxicity of TBT in iPSCs. There seems to be line-to-line differences in hiPSCs^45^. Although we used 253G1 cells in the present studies, line-to-line differences of hiPSCs in the neural differentiation should be investigated in future. Recently, significant progress has been made in the induction of differentiation of pluripotent stem cells into a variety of cell types^46^. Further studies are needed to evaluate the developmental effects of TBT on various types of iPSC-derived cells. Moreover, we showed that the TBT-induced cytotoxicity was likely caused by Mfn1-mediated mitochondrial dysfunction, which is also involved in the toxicities of other endocrine disruptive chemicals^47,48^, such as chlorpyrifos^32^ and silver nanoparticles^49^. Thus, further investigation of mitochondrial functions influenced by Mfn1 would be an important next step to examine the mechanisms underlying the toxicities induced by chemicals.

## Methods

### Chemicals and Reagents

TBT was obtained from Tokyo Chemical Industry (Tokyo, Japan) and dissolved in dimethyl sulfoxide (DMSO). Rosiglitazone (RGZ), 2-mercaptoethanol (2-ME), and sodium butyrate (NaB) were obtained from Sigma-Aldrich (St. Louis, MO, USA). Y-27632, SB431542, LDN193189, and CHIR99021 were obtained from Wako (Tokyo, Japan). The penicillin-streptomycin mixture (PS) was obtained from Thermo Fisher Scientific (Waltham, MA, USA). U0126 was obtained from Enzo Life Sciences (Farmingdale, NY, USA). All other reagents were of analytical grade and were obtained from commercial sources.

### Cell culture

We used the human iPSC line 253G1 (Riken BRC Cell Bank, Tsukuba, Ibaraki, Japan), which was established through the retroviral transduction of *Oct3/4*, *Sox2*, and *Klf4* into adult human dermal fibroblasts^50^. The cell culture was performed as previously reported^11^. Briefly, the cells were cultured under feeder-free conditions using human embryonic stem cell (ESC)-qualified Matrigel (BD Biosciences, San Jose, CA, USA) and TeSR-E8 medium (Stemcell Technologies, Vancouver, BC, Canada) at 37 °C in an atmosphere containing 5% CO_2_. For passaging, the iPSC colonies were dissociated into single cells using Accumax (Innovative Cell Technologies, San Diego, CA, USA) and cultured in TeSR-E8 medium supplemented with the ROCK-inhibitor Y-27632 (10 μM) for the first two days.

### Neural differentiation

The neuronal lineages derived from the ectodermal germ layer were induced using the dual SMAD inhibition protocol as previously described with modifications^28^. Briefly, iPSC colonies were dissociated into single cells with Accumax. The cells were seeded at a density of 7 × 10^4^ cells/cm^2^ in the TeSR-E8 medium on Matrigel-coated plates to reach a near confluent level within 2 days after seeding. The initial ectoderm differentiation was performed using a knockout serum replacement (KSR) medium [Knockout DMEM (Thermo Fisher Scientific) containing KSR (Thermo Fisher Scientific), L-glutamine (Thermo Fisher Scientific), non-essential amino acids (NEAA; Thermo Fisher Scientific), 2-ME, PS] containing SB431542 (TGFβ inhibitor, 10 μM) and LDN193189 (BMP inhibitor, 1 μg/ml). After 4 days, N2 medium [Neurobasal containing N2 (Thermo Fisher Scientific), B27 (minus vitamin A, Thermo Fisher Scientific), GlutaMAX (Thermo Fisher Scientific), PS] was added to the KSR medium with LDN193189 every 2 days.

### Mesoderm Induction

For the induction of the mesodermal germ layer, a cardiomyocyte differentiation protocol was used as previously described with modifications^24^. Briefly, iPSC colonies were dissociated into single cells using Accumax. The cells were seeded at a density of 6 × 10^4^ cells/cm^2^ in the TeSR-E8 medium on Matrigel-coated plates to achieve an approximately 80–90% confluence within 2 days after seeding. Then, the medium was replaced with RPMI1640 (Nacalai Tesque, Kyoto, Japan) containing B27 (minus insulin, Thermo Fisher Scientific), CHIR99021 (a GSK3 inhibitor, 10 μM), and PS for 1 day.

### Endoderm Induction

For the induction of endodermal germ layer, hepatic differentiation protocol was used as previously described with modifications^25^. Briefly, iPSC colonies were dissociated into single cells with Accumax. The cells were seeded at a density of 5 × 10^4^ cells/cm^2^ in the TeSR-E8 medium on Matrigel-coated plates to reach an approximately 70% confluence level within 2 days after seeding. Then, the medium was replaced with RPMI1640 containing B27 (Thermo Fisher Scientific), activinA (100 ng/ml; R&D Systems, Minneapolis, MN, USA), Wnt3a (50 ng/ml; R&D Systems), and PS. The next day, NaB (0.5 mM) was added to the culture medium, followed by a 1-day incubation period and then the medium was replaced with RPMI1640 containing B27, activinA, Wnt3a, and PS for an additional 2 days.

### Real-Time PCR

Total RNA was isolated from iPSCs using TRIzol reagent (Thermo Fisher Scientific), and quantitative real-time reverse transcription (RT)-PCR was performed using a QuantiTect SYBR Green RT-PCR kit (Qiagen, Valencia, CA, USA) using an ABI PRISM 7900HT sequence detection system (Applied Biosystems, Foster City, CA, USA) as previously reported^51^. Relative changes in transcript levels were normalized to the mRNA levels of *GAPDH*. The following primer sequences were used for real-time PCR analysis: *OTX2*, forward, 5′-ACAAGTGGCCAATTCACTCC-3′ and reverse, 5′-GAGGTGGACAAGGGATCTGA-3′; *IRX1*, forward, 5′-CGCGGATCTCAGCCTCTTC-3′ and reverse, 5′-CCCCAGGGTTGTCCTTCAGT-3′; *BRACHYURY*, forward, 5′-TGCTTCCCTGAGACCCAGTT-3′ and reverse, 5′-GATCACTTCTTTCCTTTGCATCAAG-3′; *MIXL1*, forward, 5′-CCGAGTCCAGGATCCAGGTA-3′ and reverse, 5′-CTCTGACGCCGAGACTTGG-3′; *SOX17*, forward, 5′-CGCTTTCATGGTGTGGGCTAAGGACG-3′ and reverse, 5′-TAGTTGGGGTGGTCCTGCATGTGCTG-3′; *FOXA2*, forward, 5′-TGGGAGCGGTGAAGATGGAAGGGCAC-3′ and reverse, 5′-TCATGCCAGCGCCCACGTACGACGAC-3′; *Nestin*, forward, 5′-GGCAGCGTTGGAACAGAGGT-3′ and reverse, 5′-CATCTTGAGGTGCGCCAGCT-3′; *GAPDH*, forward, 5′-GTCTCCTCTGACTTCAACAGCG-3′ and reverse, 5′-ACCACCCTGTTGCTGTAGCCAA-3′.

### Immunocytochemistry

Cell staining was performed as previously described^51^. Briefly, cells were cultured on glass coverslips, fixed in 4% paraformaldehyde in phosphate-buffered (PBS, pH 7.4) for 15 min at room temperature, and then incubated with anti- PAX6 polyclonal antibodies (1:100, Biolegend, San Diego, CA, USA) overnight at 4 °C. Then, the cells were incubated with Alexa 555-conjugated secondary antibodies (1:200, Thermo Fisher Scientific) for 1 h at room temperature. Nuclei were counterstained with DAPI (Nacalai Tesque). Fluorescence images were obtained using a BIOREVO BZ-9000 fluorescent microscope (Keyence, Osaka, Japan). PAX6 positive nuclei were counted using the ImageJ software (NIH, Bethesda, MD, USA).

### Gene Knockdown Using Short Hairpin RNA (shRNA)

Knockdown experiments were performed using Mfn1 shRNA lentiviruses from Sigma-Aldrich (MISSION shRNA), as previously reported^8^. A scrambled hairpin sequence was used as the negative control. Briefly, the cells were infected with the viruses at a multiplicity of infection (moi) of 1 in the presence of 8 μg/mL hexadimethrine bromide (Sigma-Aldrich) for 24 h. After the medium exchange, the cells were selected with 1 μg/mL puromycin for 24 h and cultured for an additional 72 h prior to the functional analyses.

### Western Blot Analysis

Western blot analysis was performed as previously reported^52^. Briefly, the cells were lysed with cell lysis buffer (Cell Signaling Technology, Danvers, MA, USA), the proteins were separated using sodium dodecyl sulfate-polyacrylamide gel electrophoresis (SDS-PAGE), and then electrophoretically transferred to Immobilon-P membranes (Millipore, Billerica, MA, USA). Then, the membranes were probed with anti-ERK1/2 polyclonal antibodies (1:1000, Cell Signaling Technology), anti-phospho ERK1/2 (Thr202/Tyr204) monoclonal antibodies (1:2000, BD Biosciences), anti-Mfn1 monoclonal antibodies (1:1000, Cell Signaling Technology), and anti-β-actin monoclonal antibodies (1:5000, Sigma-Aldrich). The membranes were then incubated with secondary antibodies against rabbit or mouse horseradish peroxidase-conjugated immunoglobulin G (IgG, Cell Signaling Technology). The bands were visualized using an enhanced chemiluminescence (ECL) western blotting analysis system (GE Healthcare, Buckinghamshire, UK) and the images were acquired using an LAS-3000 imager (FUJIFILM UK Ltd., Systems, Bedford, UK).

### Statistical analysis

All the data are presented as means ± standard deviation (SD) from three independent experiments and the Student’s *t*-test was used to analyze the data in Figs 1, 2c, 3a,c and S1. An analysis of variance (ANOVA) followed by the Bonferroni posthoc test was used to analyze the data in Figs 2b, 3b and 4. *P*-values < 0.05 were considered statistically significant.

## Electronic supplementary material

Supplementary Information
