# Supplementary material for: Tributyltin Inhibits Neural Induction of Human Induced Pluripotent Stem Cells
Source: Sci Rep. 2018 Aug 14;8:12155. doi: 10.1038/s41598-018-30615-2 (PMC6092327; doi:10.1038/s41598-018-30615-2)
Supplement: Supplementary file 1 — Supplementary Information [file 41598_2018_30615_MOESM1_ESM.pdf]

<u>**Supplementary Information**</u>

Tributyltin Inhibits Neural Induction of Human Induced Pluripotent Stem Cells

Shigeru Yamada[1,2], Yusuke Kubo[1], Daiju Yamazaki[1], Yuko Sekino[1], Yoko Nomura[3], Sachiko Yoshida[4], and Yasunari Kanda[1,]*

[1]Division of Pharmacology, National Institute of Health Sciences, Kanagawa, Japan, [2]Pharmacological Evaluation Institute of Japan (PEIJ), Kanagawa, Japan, [3]Department of Psychology, Queens College and The Graduate Center, New York, USA, [4]Department of Environmental and Life Sciences, Toyohashi University of Technology, Toyohashi, Japan

*Correspondence and requests for materials should be addressed to Y.Ka. (kanda@nihs.go.jp)

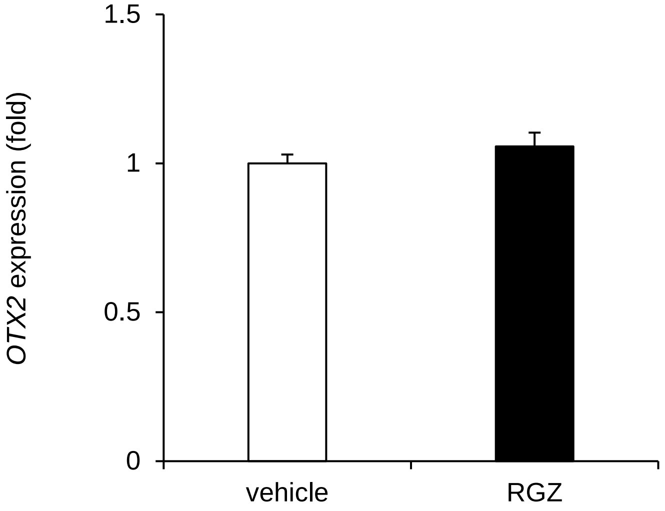

Figure S1. RGZ has a little effect on ectoderm induction of iPSCs. Ectoderm induction was initiated after exposure to 100 nM RGZ for 24 h. The cells were continuously exposed to RGZ throughout the induction. At day 2 after the induction with RGZ, *OTX2* expression was examined using real-time PCR analysis. Data are represented as means ± SD (n = 3).

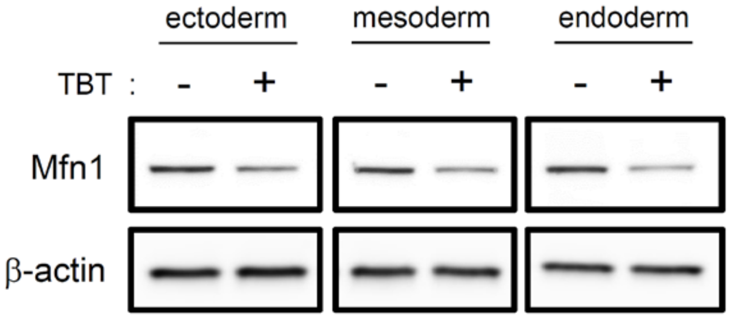

Figure S2. TBT negatively regulates Mfn1 expression. After the differentiation of iPSCs into three germ layers with TBT, the expression of Mfn1 was analyzed by western blotting using anti-Mfn1 antibodies. β-actin was used as loading control. Cropped blots were shown and the full-length blots were indicated in Supplementary Fig. 3.

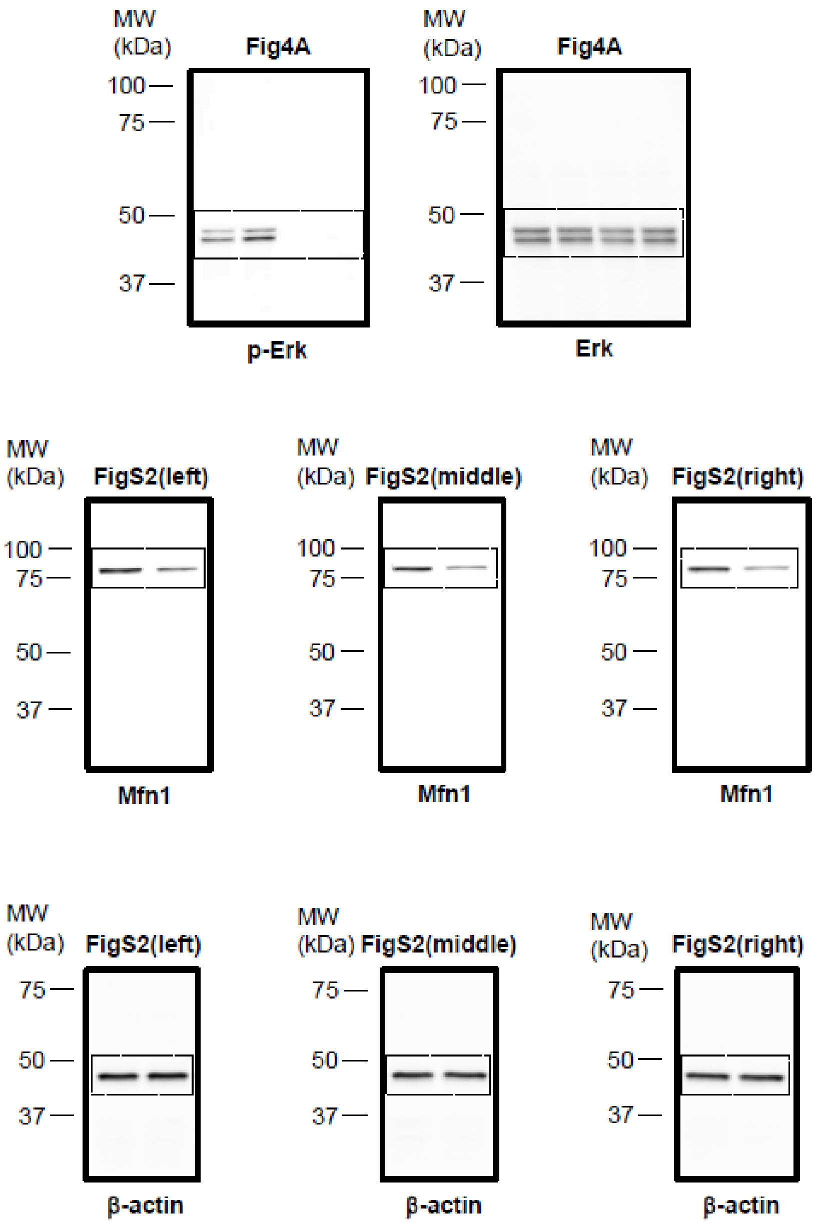

Figure S3. Images of full length blots. Dotted lines indicate cropped images used in the figures.
